# Cutaneous metastases from solid neoplasms – Literature review^☆^

**DOI:** 10.1016/j.abd.2022.10.009

**Published:** 2023-05-02

**Authors:** Bruno de Castro e Souza, Denis Miyashiro, Marcella Soares Pincelli, José Antonio Sanches

**Affiliations:** Department of Dermatology, Hospital das Clínicas, Universidade de São Paulo, São Paulo, SP, Brazil

**Keywords:** Immunohistochemistry, Medical oncology, Neoplastic metastasis

## Abstract

Cutaneous metastases from solid tumors are uncommon events in clinical practice. Most of the time, the patient already has the diagnosis of a malignant neoplasm when the cutaneous metastasis is detected. However, in up to one-third of cases, cutaneous metastasis is identified before the primary tumor. Therefore, its identification may be essential for starting treatment, although it is usually indicative of poor prognosis. The diagnosis will depend on clinical, histopathological, and immunohistochemical analysis. Sometimes the identification of the primary site is difficult; however, a thorough analysis using imaging tests and constant surveillance is important.

## Introduction

Cutaneous metastases from solid tumors are uncommon events in clinical practice. It is estimated that between 0.7% and 9% of metastases from these tumors occur in the skin.^1^

Most of the time, the patient already has a diagnosis of malignant neoplasm when the cutaneous metastasis is detected. However, in up to one-third of cases, skin metastasis is identified before the primary tumor.^2^ In some cases, the identification of the cutaneous metastasis indicates cancer recurrence, and in 79% of cases, there are concurrent visceral metastases.^3^ The clinical presentation is diverse and can simulate inflammatory diseases, benign tumors, or even malignant skin tumors. This review discusses the current paradigm of metastasis mechanisms and displays the main clinical presentations. For didactic reasons, clinical presentations will be discussed according to the primary tumor site, including the ones originally from the skin. Paraneoplastic manifestations and hematological malignancies will not be reviewed in this article.

## Etiopathogenesis

Metastasis is defined as the development of a tumor at some distance from the primary site. It develops predominantly by vascular invasion (lymphatic or hematogenous). There is a discussion in the literature whether implants should be considered as actual metastases either by contiguity or iatrogenically. Currently, the metastatic process is understood as a complex phenomenon that occurs in parallel with the development of the primary tumor. Due to its greater importance, metastases through vascular invasion will be discussed in more detail.

The pathophysiology of metastasis is divided into six stages: (1) local invasion, (2) intravasation, (3) survival in the circulation, (4) arrest in a distant organ, (5) extravasation, (6) micrometastasis formation and metastatic colonization.^4^

### Local invasion

After the development of the malignant tumor in the primary site, local invasion occurs. This event consists of the entry of malignant cells into the soft tissue adjacent to the tumor. For this to occur, disruption of the basement membrane secondary to the action of matrix metalloproteinases is necessary.^4^

### Intravasation

Intravasation is a complex phenomenon that allows malignant cells to enter the blood or lymphatic vessels. This process is essential for malignant cells to reach distant sites and continue the metastatic process. It has been well-described that malignant tumors have the ability to produce neoangiogenesis.^5^ What happens is that the neoformed vessels have little coverage of pericytes and endothelial cells with little cell adhesion, facilitating the entry of malignant cells (intravasation).^4^, ^5^ Curiously, more recent evidence demonstrates that this phenomenon occurs predominantly during sleep.^6^

### Survival in circulation

Once inside the vessels, the malignant cells need to survive. They are subject to trauma resulting from hemodynamic forces and, in addition, are subject to the immune system action. The main adaptive way to evade these mechanisms is the formation of platelet microthrombi around the neoplastic cells, which protect them from these phenomena.^4^

### Arrest in a distant organ

Tumors can reach virtually any tissue in the body through blood or lymphatic circulation. The major question is why some tumors have preferential metastasis sites.^7^ For instance, there is a clear preference for bone in prostate cancer metastases or, in cases of colon tumors, metastases to the liver.^8^ A strong interaction between the primary tumor and the target site soft tissue is believed to be necessary.^4^ Although the skin is a highly vascularized organ, few tumors, such as melanoma, have an obvious predilection for it. Perhaps this is due to good immunological protection or low soft tissue-malignant tumor interaction.

### Extravasation

After reaching the target organ circulation, malignant cells must exit the vessels to reach the soft tissue. This process may occur due to mechanical vascular rupture or a more complex process. In the latter case, tumor cells might induce vascular hyperpermeability secondary to the release of proteins such as epiregulin (EREG), cyclooxygenase 2 (COX-2), metalloproteinase 1 and 2 (MMP-1 and MMP-2).^9^ It is important to emphasize that each protein has a specific action on the vascularization of each target organ, reinforcing the idea that each malignant tumor has one or more preferential metastasis sites.^4^

### Micrometastasis formation and metastatic colonization

The last step is tumor survival in the target site soft tissue. For this to occur, the tumor needs to have a self-renewal and proliferation capacity. Additionally, it releases substances that make the adjacent soft tissue more receptive to its multiplication.^4^ Micrometastases may persist in a latent state for months or years. During this period, micrometastases that acquire genetic or epigenetic alterations and can make the microenvironment more favorable to their development with the formation of macroscopic metastases with significant lethal potential.^4^

## Epidemiology

There is divergence in the literature about which tumors most frequently metastasize to the skin. More recent studies indicate that melanoma is the most frequent one, but in some classic studies, this neoplasm appears only in the fourth place.^10^, ^11^ A 2002 meta-analysis identified that breast tumors are the most frequent ones (24%) followed by kidney (4%), ovary (3.8%), bladder (3.6%), lung (3.4%), colorectal (3.4%), and prostate (0.7%) cancer.^12^ However, cases of melanoma were excluded from this study.

There is an epidemiological difference according to sex. A Brazilian study with 209 cases of cutaneous metastases identified that the most frequent cutaneous metastases in women originated from breast (63.19%), large intestine (10.41%), and lung cancer (4.16%) whereas in men, the order of frequency was lung, stomach, and larynx (33.84%, 12.3%, and 7.69%, respectively).^13^ When specifically analyzing the pediatric population, the most frequent primary tumors are rhabdomyosarcoma and neuroblastoma.^14^

It is a well-known fact that there are some areas of the skin where the appearance of metastatic lesions is more frequent. The anterior thorax is the most common site, followed by the abdomen, head and neck (including the scalp), and limbs.^11^, ^12^, ^13^ There is a clear predilection for the affected site according to the primary tumor; therefore, the cutaneous metastasis site may indicate a possible neoplastic origin. For instance, breast cancer most often metastasizes to the anterior thorax, whereas gastrointestinal tract tumors secondarily most often affect the abdomen.^15^ Table 1 summarizes the preferred sites of skin metastases.

**Table 1:** Main locations of cutaneous metastases according to the primary tumor^15^

Cutaneous site	Primary tumor
Scalp	Breast, lung, kidney
Face and neck	Squamous cell carcinoma of the head and neck, kidney, lung
Anterior thorax	Breast, lung, melanoma
Abdomen	Colon, stomach, lung, ovary
Umbilical region	Stomach, pancreas, colon, ovaries, kidney, breast
Pelvis	Colon, bladder

## Clinical presentation

### Skin

Several originally cutaneous malignant neoplasms can develop metastases to the skin. Of these, the most frequent is melanoma and, for that reason, it will be discussed in more detail. However, it is important to emphasize that squamous cell carcinoma, Merkel cell carcinoma, and even malignant pilomatricoma can develop skin metastases.^11^

A North-American study has shown that the number of cutaneous metastases from melanoma has been increasing in recent years.^16^ This is not a rare event in the natural history of the disease. Secondary skin involvement occurs in 10% to 17% of patients with melanoma and is present in up to 50% of individuals with disseminated disease.^17^ In most cases, the skin is the first site of metastatic involvement and, in about 30% of cases, it occurs after lymph node metastasis.^18^ The concomitant finding of cutaneous, nodal, and visceral metastasis occurs in approximately 10% of cases.^18^

Cutaneous melanoma metastases are divided into microsatellite, satellite, in-transit or distant metastases. The first is microscopic metastasis (cutaneous or subcutaneous) disconnected from the primary tumor, therefore, it is not perceived in the clinical examination. A satellite metastasis is a metastatic tumor visible up to 2 cm from the primary tumor site. In-transit metastasis is defined as any cutaneous or subcutaneous metastasis that is more than 2 cm away from the primary lesion but within the regional lymphatic drainage.^19^ Despite this differentiation, both satellite lesions and in-transit metastasis correspond to intralymphatic spread and have similar therapeutic and prognostic implications.^20^ It is also important to differentiate lymph node metastases from local recurrence that occurs as a result of incomplete resection of the primary tumor.

Distant metastasis is related to hematogenous spread and occurs when the cutaneous metastatic site is far from the lymphatic drainage focus of the primary tumor. This differentiation is important, as in the first two scenarios the patient is considered to have locoregional disease, whereas, in the latter, the patient has distant metastasis, which considerably worsens the patient’s prognosis.^19^ Melanoma staging is beyond the scope of this article and therefore will not be discussed.

**Figure 1:**
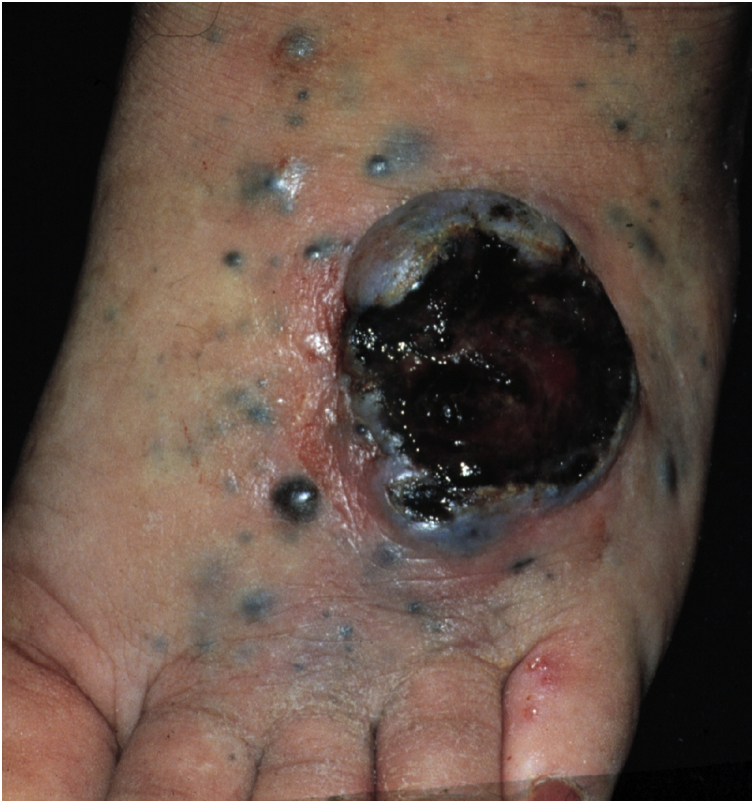
Patient with cutaneous metastases of melanoma with an ulcerated tumor lesion and several papules and satellite nodules on the foot

Cutaneous metastases from melanoma are most commonly found on the back in men and on the lower limbs in women. The fact that secondary cutaneous locations occur in the same anatomical location as the initial cutaneous location in more than 30% of cases explain the distinct patterns of metastases between sexes.^18^ Clinically, there are generally black or brownish dermal or subcutaneous papules or nodules that may ulcerate (Fig. 1).^21^ Eventually, they can be amelanotic and reach large dimensions, leading to significant morbidity.^16^ In the case of in-transit metastases, they are usually located between the primary tumor site and the regional lymph node. However, in some situations, they may arise on the opposite side, due to a change in lymphatic flow secondary to the tumor presence. ^20^ More atypical cases, such as erythematous plaques (erysipela-like), sclerosing (*en cuirasse*), purpuric, and telangiectatic lesions have been described.^22^ There are also reports of zosteriform metastases with vesiculobullous lesions and nodules distributed across a dermatome.^23^

### Breast

Most cases of breast cancer cutaneous metastasis are due to adenocarcinomas (77%–82%).^17^, ^24^ They usually arise in the anterior thorax due to regional lymphatic spread.^25^ Clinically, the most common form of presentation comprises normochromic papules or nodules. However, some well-characterized presentations are described, namely: telangiectatic carcinoma, erysipeloid carcinoma, carcinoma *en cuirasse* and neoplastic alopecia.^26^

**Figure 2:**
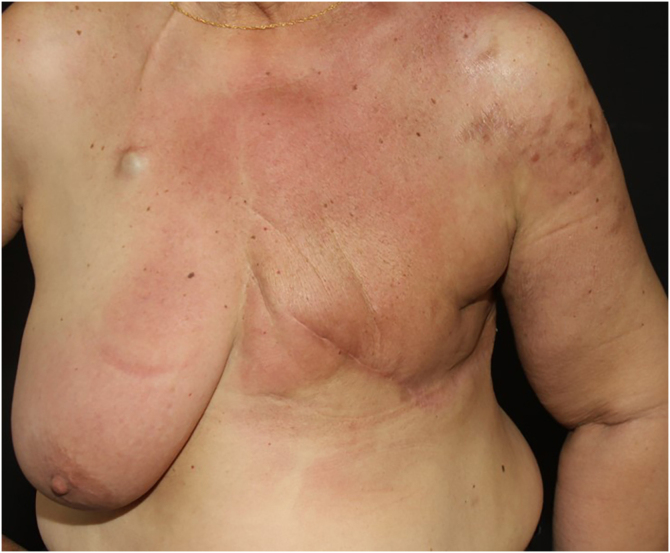
Erythematous infiltrated plaque on the chest of a patient with cutaneous metastasis of breast cancer

Telangiectatic carcinoma presents with violaceous papules on a telangiectatic surface, arising close to the previous mastectomy scar. Less commonly, papulovesicular lesions similar to circumscribed lymphangioma appear.^27^ Erysipeloid (or inflammatory) carcinoma appears as erythematous, warm, plaques with well-demarcated borders, affecting the breast and adjacent skin (Fig. 2).^26^ Marneros et. al. have shown that telangiectatic carcinoma spread occurs predominantly via blood vessels, whereas in erysipeloid carcinoma the spread is lymphatic.^27^ In carcinoma *en cuirasse*, the skin acquires an infiltrated hardened appearance, similar to scleroderma. Finally, in neoplastic alopecia, there are nodules or hardened plaques on the scalp, which result in alopecia.^26^ The alopecia can be cicatricial and irreversible, as neoplastic cells can destroy hair follicles and induce fibroplasia.^28^ As with melanoma, cases with papules arranged in a zosteriform pattern have also been described.^26^ It is noteworthy that presentations similar to telangiectatic, *en cuirasse*, erysipeloid carcinoma, and mucinous alopecia have been described in metastases from other tumors, but are more commonly secondary to breast adenocarcinoma.^21^

As for prognosis, Hu et. al. demonstrated that the mean survival of patients with breast cancer and cutaneous metastasis is 57 months, and only 25 months among those with concomitant visceral metastasis.^29^

### Lung

Lung cancer metastases are most often seen in the liver, bones, central nervous system, adrenal glands, and mediastinal lymph nodes, with cutaneous metastasis being rare.^30^ It is estimated that between 1% to 12% of patients with lung cancer have cutaneous metastases.^30^, ^31^ When analyzing all types of cutaneous metastases, patients with lung cancer metastases have the worst prognosis.^32^ Although they can affect any site on the skin, they preferentially affect the head and neck, anterior thorax, and abdomen. As with breast cancer, adenocarcinoma is the most common metastatic type, followed by squamous cell carcinoma, while small cell lung cancer is the least frequent one.^21^ The primary tumor is usually located in the upper pulmonary lobes.^30^, ^31^

**Figure 3:**
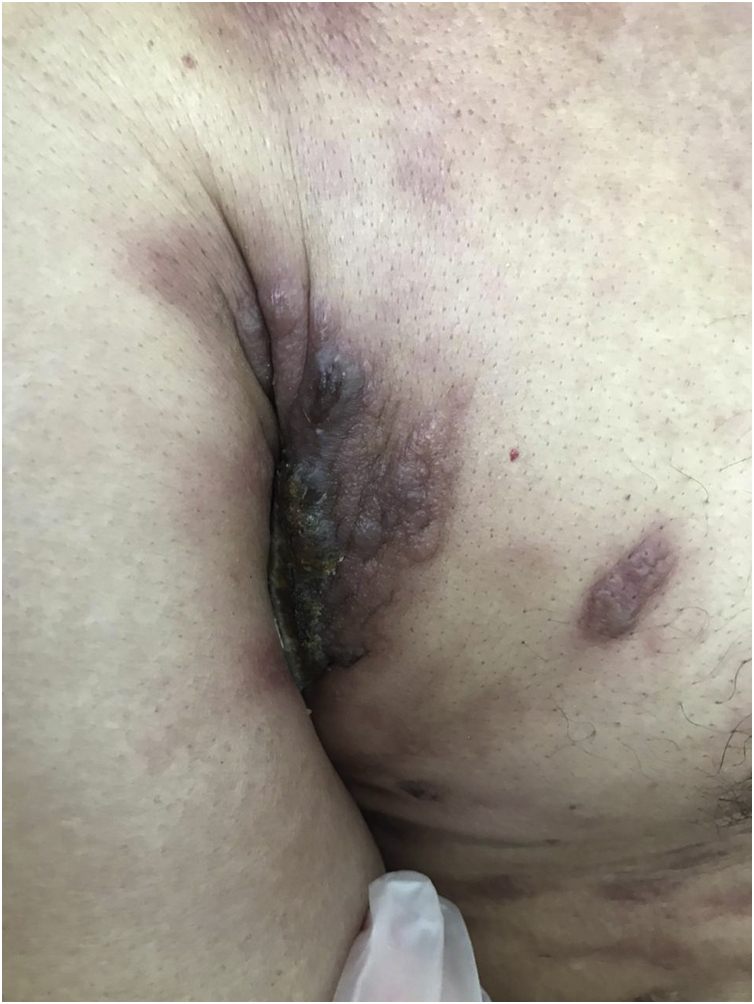
Metastasis of lung adenocarcinoma presenting as an erythematous-violaceous plaque located in the right axilla

Clinically, they are indistinguishable from metastases from tumors with other origins. They usually present as normochromic or slightly erythematous subcutaneous nodules, hardened and adhered to deep planes (Fig. 3). Most of the time they appear as solitary lesions, but multiple lesions can appear subsequently. There are reports of metastases simulating keratoacanthomas,^33^ erythematous nodules located at the tip of the nose (“clown nose”),^34^ with a zosteriform pattern of distribution or simulating erysipelas.^35^ A little-known involvement is the subungual type. A literature review showed that subungual metastasis occurs more frequently in patients with lung (41%), genitourinary (17%), and breast (9%) cancer.^36^ These metastases manifest as subungual erythematous-violaceous nodules or as edema, erythema, and pain in the distal phalanges simulating an infectious condition.^36^

It is noteworthy that most of the time when there is cutaneous metastasis, other extrapulmonary sites are often also detected.^21^

### Gastrointestinal tract

Among the malignant neoplasms of the gastrointestinal tract, colorectal adenocarcinoma is the one that most often leads to cutaneous metastases.^12^ At the time of the diagnosis, about 10% of the cases already have distant metastasis, and the most commonly affected sites are the liver, lungs and central nervous system.^37^ It is estimated that about 4% of patients with colorectal cancer progress to cutaneous metastasis, and the mean time from the diagnosis to metastasis onset is 25 months.^17^, ^38^ The mean age at the metastasis diagnosis is 55 years.^39^

Skin involvement can occur either by contiguity, lymphatic or hematogenous spread, or by spread along embryonic remnants, such as the urachus.^40^ For this reason, the skin of the abdomen is the most frequently affected cutaneous site, followed by the presacral and perineal region, with the latter being more often related to rectal cancer.^38^ In one study, most cutaneous metastases arose from the colectomy surgical incision sites.^17^ Nonetheless, there are reports of spread to the most diverse locations, such as the thorax, upper limbs, and head and neck region.^40^ When the metastasis affects the umbilical region, it is called Sister Mary Joseph’s nodule.^41^ This finding is not pathognomonic of colorectal tumors, as it has been reported in other malignant tumors such as stomach, ovary, pancreas, and even uterine cervix, gallbladder, and small bowel cancer.^41^

There is no characteristic clinical presentation, with most appearing as pink or reddish, firm nodular lesions that may ulcerate.^38^ Other described presentations are epidermal cyst-like lesions, neurofibromas, annular erythema, condylomas, elephantiasis nostra verrucosa, and areas of alopecia.^17^, ^40^ In the case of Sister Mary Joseph’s nodule, there are typically firm nodules measuring 0.5 to 2 cm, which may eventually show purulent, serous, or bloody secretion.^21^

In the case of gastric tumors, signet-ring cell carcinoma is the most common to show cutaneous metastasis.^42^ As with colorectal adenocarcinoma, the abdomen is the most commonly affected site and clinically may present as nodules, erysipela-like lesions, or epidermal cyst-like lesions.^42^ The prognosis is very poor in both metastases, with a mean survival of a few months.

Finally, there have been rare reports of cutaneous metastases from malignant neoplasms of the esophagus, pancreas, and liver.^43^ In these cases, the lesions were identified in different sites, such as the scalp, abdomen, and dorsum region.

### Genitourinary system

Of the cancers of the urinary system, renal cancer is the most common one. As renal cell carcinoma displays few signs and symptoms, most are diagnosed in later stages when metastases already exist.^44^ In these metastatic cases, the skin is the site of the metastasis less than 2% of the time, with clear cell renal carcinoma being the most common histopathological subtype.^21^ In most cases, the patient already has a diagnosis of renal cancer when the cutaneous metastasis is identified (six to five years after the diagnosis), but in up to 20% of cases, the cutaneous lesion appears before the identification of the primary renal tumor.^45^

The head and neck region is frequently affected by metastases from renal cell carcinoma. The current theory explains that renal veins have anastomoses with the vertebral plexuses, which in turn are connected to the cephalic vasculature. This would allow the hematogenous spread of renal neoplastic cells to the head and neck region.^46^ It is important to emphasize that renal cell carcinoma, as well as follicular thyroid carcinoma and hepatocellular carcinoma preferentially show hematogenous spread.^46^

Cutaneous metastases from renal cell carcinoma present as rapidly growing nodules that may be normochromic or more characteristically reddish. This is due to high tumor vascularity and therefore can be confused with hemangiomas, ruby angiomas, or pyogenic granulomas.^47^ The presence of metastasis is a marker of worse prognosis and survival expectancy is about six months.

More rarely, cases of cutaneous metastasis of bladder urothelial carcinoma are described with a mean survival of fewer than 12 months. In most cases, cutaneous involvement occurs through direct tumor invasion, but it may also be secondary to vascular spread (lymphatic or hematogenous) or iatrogenic implantation (following procedures such as cystectomy).^48^ Therefore, in most cases, the lesion is located in the lower abdomen, pelvis or scrotum. The clinical presentation is non-specific and may be as single or multiple nodules, infiltrated plaques, or even showing a sclerosing appearance.^49^ Finally, Savell et. al. described a case of urothelial carcinoma manifesting as livedo racemosa due to vascular occlusion.^50^

Cutaneous metastases from gynecological (excluding breast) tumors may account for up to 8.5% of secondary implants in female patients.^43^ Most cases are derived from malignant ovarian neoplasms, but they can also originate from uterine cervix cancer. For anatomical reasons, the abdomen (especially the umbilicus) is the most commonly affected site.^43^ As for males, it is known that prostate cancer, which is very frequent, rarely courses with cutaneous metastases.^11^

### Head and neck

Head and neck squamous cell carcinomas have the lung and bones as the most common sites of distant metastasis. Pitman et. al., in a cohort of 2,491 patients with head and neck squamous cell carcinoma, identified only 19 (0.763%) with cutaneous metastasis.^51^ Yoskovitch et al. al. identified 19 cases (2.4%) of cutaneous metastases among 798 cases of head and neck squamous cell carcinoma.^52^ It is important to note that in these studies, cases of cutaneous involvement due to tumor contiguity were excluded, and only those with distant metastasis were included.

Squamous cell carcinomas of the oral cavity, particularly the mouth floor, are the ones that most often lead to cutaneous metastases.^52^ The neck, scalp, and anterior thorax are the sites affected in 63%, 15%, and 10% of cases, respectively, and the lesions are multiple in most cases.^52^ The mean survival after the onset of metastasis is only three months.^51^

**Figure 4:**
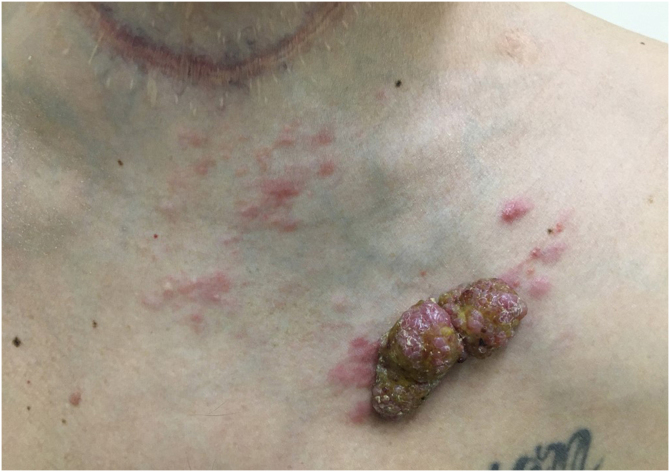
Tumor lesion with papules and satellite erythema on the anterior thorax in a patient with cutaneous metastasis of thyroid cancer. Note the previous thyroidectomy scar

Another frequent malignant tumor of the neck is thyroid carcinoma, the most common endocrinological malignancy.^53^ The two main histopathological types (papillary and follicular) most frequently affect the skin secondarily, with an estimated incidence of less than one in 1,000 cases.^54^, ^55^ There are very rare cases of cutaneous metastasis from anaplastic or medullary carcinomas.^56^ It occurs in men and women in equal proportions, with a mean age of 50 years.^54^ The scalp is involved in most cases, although the involvement of other areas such as the trunk (anterior and posterior), face, and neck have also been reported (Fig. 4).^54^, ^57^ There is a characteristic clinical presentation since most reports in the literature describe erythematous, pruritic, or ulcerated nodules located on the scalp.^53^, ^57^ The mean survival after the diagnosis of cutaneous metastasis is 19 months.^54^

## Diagnosis

Definitive diagnosis depends on a thorough clinical examination, imaging tests, and histopathological analysis. Table 2 summarizes histopathological evidence for suspected skin metastases.

**Table 2:** Histopathological clues for suspected cutaneous metastases^58^

Presence of neoplastic cells within lymphatic or blood vessels.
Nodules located in the reticular dermis and/or hypodermis without contact with the epidermis (grenz zone).
Presence of neoplastic cells lined up between collagen fibers (“single filing”)

**Figure 5:**
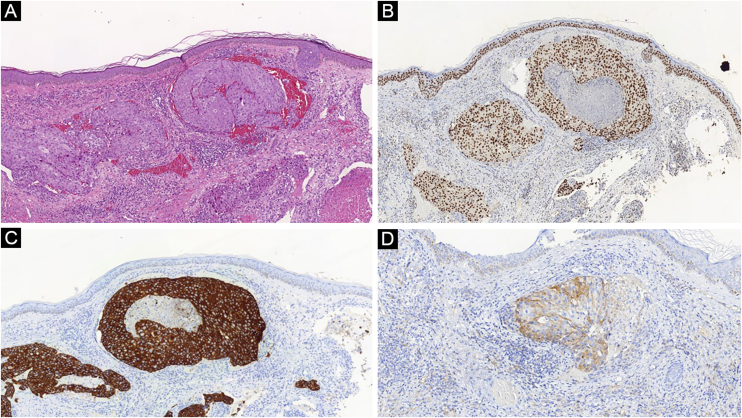
(A) Atypical cell nodules inside dermal capillaries in a case of breast adenocarcinoma metastasis (Hematoxylin & eosin, ×100). (B) Positive GATA3, (C) CK7 and (D) mammaglobin corroborate the diagnosis

**Figure 6:**
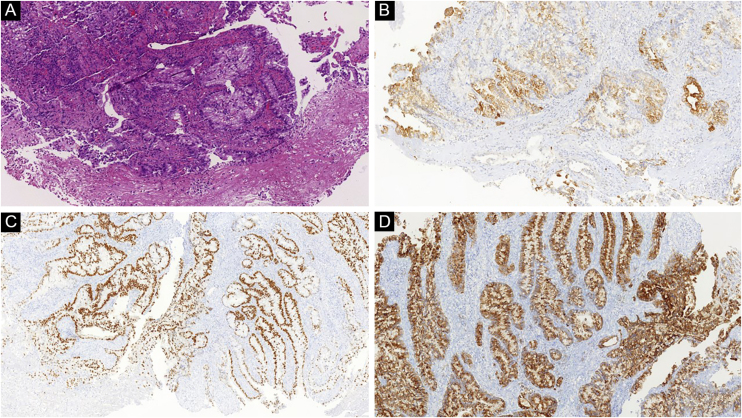
(A) Atypical nodular proliferation in the deep dermis in a patient with cutaneous metastasis of colon adenocarcinoma (Hematoxylin & eosin, ×100). (B) Positive CK7, (C) CDX2 and (D) EMA corroborate the diagnosis

Histopathological and immunohistochemical characteristics of cutaneous metastases tend to be similar to that of the primary tumor; however, metastatic cells tend to be more anaplastic, that is less differentiated.^58^ Hussein recommends a basic immunohistochemical panel including CD45 (for lymphoid malignancies), AE1/AE3 pankeratins (for most carcinomas), S100 (melanomas), and CD34 (vascular neoplasms and leukemias). Subsequently, a second panel may be performed including lymphoid markers (CD3 and CD20 for T and B lymphocytes, respectively), epithelial markers (EMA and CEA), chromogranin (neuroendocrine tumors), thyroid transcription factor (lung cancer), WT1 (ovarian carcinoma), prostate-specific antigen and acid phosphatase (prostate carcinoma).^58^ Table 3 summarizes the immunophenotypes of the main tumors that metastasize to the skin and Figure 5, Figure 6 exemplify them.

**Table 3:** Summary of immunophenotypes of the main tumors that metastasize to the skin.^58^

Primary tumor	Positive immunohistochemical markers	Negative immunohistochemical markers
Breast adenocarcinoma	CK7, Estrogen and Progesterone Receptor, Ber-EP4, GATA3, Mammaglobin	CK20, CK5/6
Colon adenocarcinoma	CK20, CDX2, CEA.	CK7 (some cases may be positive)
Stomach adenocarcinoma	CK20, CK7, CEA, CDX2	CK7 (some cases may be positive)
Lung adenocarcinoma	FTT-1, CK7, CEA, EMA	CK5/6, CK20
Small-cell lung cancer	FTT-1, neuron-specific enolase (NSE), chromogranin, synaptophysin	CK7, CK20, CD99
Clear cell renal carcinoma	CAM5.2, EMA, CD10, RCC-Ma, vimentin, S100	Melan-A, TTF-1, CK7, CK20
Melanoma	Vimentin, S100, tyrosinase, Melan-A, HMB45, MITF	–
Bladder/urothelial	P63, CK5/6, CK7, CK20, uroplakin III	–
Prostate	PSA, prostatic acid phosphatase,	CK7, CK20, thrombomodulin
Ovary	CA.125, CK7, PAX8	CK20 (except some mucinous variants)
Head and neck squamous cell carcinoma	CK5/6, p63, CK903	–
Thyroid	FTT-1, thyroglobulin, PAX8	–

Even after immunohistochemical evaluation, sometimes the pathologist cannot accurately determine the primary tumor and only classifies it into general classes, such as adenocarcinoma, squamous cell carcinoma, or undifferentiated carcinoma.^15^ In some cases, after the initial evaluation, the primary origin of the tumor cannot be determined and it is then classified as cancer of an unknown primary site.^59^ In these cases, a more in-depth assessment is required. The first radiological exams to be performed are computed tomography (CT) and/or magnetic resonance imaging (MRI) of different segments of the body, associated with mammography when breast cancer is suspected. If these are inconclusive, the next step is the combined positron emission tomography (PET) and CT (PET-CT) imaging test.^59^

## Treatment

Overall, treatment should be directed at the primary tumor. As in most cases, the presence of cutaneous metastasis indicates advanced disease, systemic antineoplastic therapy is usually the chosen therapeutic option. However, systemic therapy may have reduced efficacy in cutaneous lesions, and skin-directed therapies have an adjuvant function.

A meta-analysis including 47 studies and 4,313 skin metastases evaluated five skin-directed therapies: electrochemotherapy, photodynamic therapy, radiotherapy, intralesional therapy, and topical therapy.^60^ The researchers found a complete response rate and an objective response rate of 35.5% and 60.2%, respectively. Electrochemotherapy for cutaneous metastases uses short electrical pulses directed at the tumor to permeabilize cell membranes aiming to increase the absorption of intralesional or intravenous chemotherapy.

As for intralesional therapies, there have been studies with injections of recombinant antibodies directed at ErbB2/HER2 in cases of uterine cervix and breast cancer and interferon and interleukin-2 in cases of melanoma.^61^ It is also possible to perform treatment with topical immunotherapy for some tumors. A Brazilian study showed that diphencyprone can lead to up to 37% of complete response in cases of cutaneous melanoma metastases.^62^

## Conclusion

Although infrequent, cutaneous metastasis can be the first sign of a malignant neoplasm or indicate its recurrence. The dermatologist must know how to identify suspicious lesions and perform an adequate biopsy. The diagnosis will depend on the clinical, histopathological, and immunohistochemical analysis. Sometimes the identification of the primary site is difficult, but a thorough evaluation using imaging tests and constant surveillance is important.

## Financial support

None declared.

## Authors’ contributions

Bruno de Castro e Souza: Drafting and editing of the manuscript; critical review of the literature.

Denis Miyashiro: Collection, analysis and interpretation of data; drafting and editing of the manuscript; critical review of the literature.

Marcella Soares Pincelli: Design and planning of the study, drafting and editing of the manuscript; critical review of the literature.

José Antonio Sanches: Design and planning of the study, drafting and editing of the manuscript; critical review of the literature.

## Conflicts of interest

None declared.

## References

[1] Llancapi P., Gutiérrez R., Paiva O. (1996). Cutaneous metastases. Clinical pathological review. Rev Med Chil.

[2] Schwartz R.A. (1995). Cutaneous metastatic disease. J Am Acad Dermatol.

[3] Teyateeti P., Ungtrakul T. (2021). Retrospective review of cutaneous metastasis among 11,418 patients with solid malignancy: a tertiary cancer center experience. Medicine (Baltimore)..

[4] Valastyan S., Weinberg R.A. (2011). Tumor metastasis: molecular insights and evolving paradigms. Cell.

[5] Carmeliet P., Jain R.K. (2011). Principles and mechanisms of vessel normalization for cancer and other angiogenic diseases. Nat Rev Drug Discov.

[6] Diamantopoulou Z., Castro-Giner F., Schwab F.D., Foerster C., Saini M., Budinjas S. (2022). The metastatic spread of breast cancer accelerates during sleep. Nature.

[7] Friedl P., Wolf K. (2003). Tumour-cell invasion and migration: diversity and escape mechanisms. Nat Rev Cancer.

[8] Auguste P., Fallavollita L., Wang N., Burnier J., Bikfalvi A., Brodt P. (2007). The host inflammatory response promotes liver metastasis by increasing tumor cell arrest and extravasation. Am J Pathol.

[9] Padua D., Zhang X.H., Wang Q., Nadal C., Gerald W.L., Gomis R.R. (2008). TGFbeta primes breast tumors for lung metastasis seeding through angiopoietin-like 4. Cell.

[10] Brownstein M.H., Helwig E.B. (1972). Patterns of cutaneous metastasis. Arch Dermatol.

[11] Queirós C.S., Filipe P.L., Soares de Almeida L. (2020). Cutaneous metastases from solid neoplasms in the 21st century: a retrospective study from a Portuguese tertiary care center. J Eur Acad Dermatol Venereol.

[12] Krathen R.A., Orengo I.F., Rosen T. (2003). Cutaneous metastasis: a meta-analysis of data. South Med J.

[13] Sittart J.A., Senise M. (2013). Cutaneous metastasis from internal carcinomas: a review of 45 years. An Bras Dermatol.

[14] Wesche W.A., Khare V.K., Chesney T.M., Jenkins J.J. (2000). Non-hematopoietic cutaneous metastases in children and adolescents: thirty years’ experience at St. Jude Children’s Research Hospital. J Cutan Pathol.

[15] Martínez M.C.F.A., Parra-Blanco V., Izquierdo J.A.A., Fernández R.M.S. (2013). Cutaneous metastases of internal tumors. Actas Dermosifiliogr.

[16] Wong C.Y., Helm M.A., Helm T.N., Zeitouni N. (2014). Patterns of skin metastases: a review of 25 years’ experience at a single cancer center. Int J Dermatol.

[17] Lookingbill D.P., Spangler N., Helm K.F. (1993). Cutaneous metastases in patients with metastatic carcinoma: a retrospective study of 4020 patients. J Am Acad Dermatol.

[18] Savoia P., Fava P., Nardò T., Osella-Abate S., Quaglino P., Bernengo M.G. (2009). Skin metastases of malignant melanoma: a clinical and prognostic survey. Melanoma Res.

[19] Keung E.Z., Gershenwald J.E. (2018). The eighth edition American Joint Committee on Cancer (AJCC) melanoma staging system: implications for melanoma treatment and care. Expert Rev Anticancer Ther.

[20] Balch C.M., Gershenwald J.E., Soong S.J., Thompson J.F., Atkins M.B., Byrd D.R. (2009). Final version of 2009 AJCC melanoma staging and classification. J Clin Oncol..

[21] Strickley J.D., Jenson A.B., Jung J.Y. (2019). Cutaneous metastasis. Hematol Oncol Clin North Am.

[22] Sariya D., Ruth K., Adams-McDonnell R., Cusack C., Xu X., Elenitsas R. (2007). Clinicopathologic correlation of cutaneous metastases: experience from a cancer center. Arch Dermatol.

[23] Savoia P., Fava P., Deboli T., Quaglino P., Bernengo M.G. (2009). Zosteriform cutaneous metastases: a literature meta-analysis and a clinical report of three melanoma cases. Dermatol Surg.

[24] Gan E.Y., Chio M.T., Tan W.P. (2015). A retrospective review of cutaneous metastases at the National Skin Centre Singapore. Australas J Dermatol.

[25] Wong C.Y., Helm M.A., Kalb R.E., Helm T.N., Zeitouni N.C. (2013). The presentation, pathology, and current management strategies of cutaneous metastasis. N Am J Med Sci.

[26] Mordenti C., Peris K.M., Fargnoli C., Cerroni L., Chimenti S. (2000). Cutaneous metastatic breast carcinoma. Acta dermatovenerologica.

[27] Marneros A.G., Blanco F., Husain S., Silvers D.N., Grossman M.E. (2009). Classification of cutaneous intravascular breast cancer metastases based on immunolabeling for blood and lymph vessels. J Am Acad Dermatol.

[28] Scheinfeld N. (2006). Review of scalp alopecia due to a clinically unapparent or minimally apparent neoplasm (SACUMAN). Acta Derm Venereol.

[29] Hu S.C., Chen G.S., Lu Y.W., Wu C.S., Lan C.C. (2008). Cutaneous metastases from different internal malignancies: a clinical and prognostic appraisal. J Eur Acad Dermatol Venereol.

[30] Khaja M., Mundt D., Dudekula R.A., Ashraf U., Mehershahi S., Niazi M. (2019). Lung cancer presenting as skin metastasis of the back and hand: a case series and literature review. Case Rep Oncol.

[31] Batalla A., Aranegui B., de la Torre C., Prieto O. (2012). Cutaneous metastasis of lung cancer: two case reports and review of the literature. Med Cutan Iber Lat Am.

[32] Schoenlaub P., Sarraux A., Grosshans E., Heid E., Cribier B. (2001). Survival after cutaneous metastasis: a study of 200 cases. Ann Dermatol Venereol.

[33] Reich A., Kobierzycka M., Woźniak Z., Cisło M., Szepietowski J.C. (2006). Keratoacanthoma-like cutaneous metastasis of lung cancer: a learning point. Acta Derm Venereol.

[34] Camarasa A., Chiner E., Sancho J. (2009). Clown nose as an initial manifestation of squamous cell carcinoma of the lung. Arch Bronconeumol.

[35] Marcoval J., Gallego M.I., Moreno A. (2008). Inflammatory cutaneous metastasis as a first sign of recurrence of squamous cell carcinoma of the lung. Actas Dermosifiliogr.

[36] Cohen P.R. (2001). Metastatic tumors to the nail unit: subungual metastases. Dermatol Surg.

[37] Wong N.S., Chang B.M., Toh H.C., Koo W.H. (2004). Inflammatory metastatic carcinoma of the colon: a case report and review of the literature. Tumori.

[38] Yilmaz K., Atalay C. (2009). Cutaneous metastases in colorectal cancer. Trakya Univ Tip Fak Derg.

[39] Dehal A., Patel S., Kim S., Shapera E., Hussain F. (2016). Cutaneous metastasis of rectal cancer: a case report and literature review. Perm J.

[40] Bittencourt M.J.S., Imbiriba A.A., Oliveira O.A., Santos J.E.B.D. (2018). Cutaneous metastasis of colorectal cancer. An Bras Dermatol.

[41] İşcan Y., Karip B., Onur E., Özbay N., Tezer S., Memişoğlu K. (2014). Sister Mary Joseph nodule in colorectal cancer. Ulus Cerrahi Derg.

[42] Abbasi F., Abbasi A., Mahmodlou R., Mehdipour E. (2018). Cutaneous metastasis of gastric carcinoma: a rare case with unusual presentation site. Indian J Dermatopathol Diagn Dermatol.

[43] Vernemmen A.I.P., Li X., Roemen G.M.J.M., Speel E.M., Kubat B., Hausen A.Z. (2022). Cutaneous metastases of internal malignancies: a single-institution experience. Histopathology.

[44] Graves A., Hessamodini H., Wong G., Lim W.H. (2013). Metastatic renal cell carcinoma: update on epidemiology, genetics, and therapeutic modalities. Immunotargets Ther.

[45] Bujons A., Pascual X., Martínez R., Rodríguez O., Palou J., Villavicencio H. (2008). Cutaneous metastases in renal cell carcinoma. Urol Int.

[46] Errami M., Margulis V., Huerta S. (2016). Renal cell carcinoma metastatic to the scalp. Rare Tumors.

[47] Lee H.J., Lee A., Tan D., Du J., Wang Y., Tang P.Y. (2020). Cutaneous metastasis of renal cell carcinoma. Lancet Oncol.

[48] Hasan O., Houlihan M., Wymer K., Hollowell C.M.P., Kohler T.S. (2019). Cutaneous metastasis of bladder urothelial carcinoma. Urol Case Rep.

[49] Lees A.N. (2015). Cutaneous metastasis of transitional cell carcinoma of the urinary bladder eight years after the primary: a case report. J Med Case Rep.

[50] Savell A.S., Morris B., Heaphy M.R. (2020). Cutaneous metastasis of urothelial carcinoma resulting in vascular occlusion and livedo racemosa. JAAD Case Rep.

[51] Pitman K.T., Johnson J.T. (1999). Skin metastases from head and neck squamous cell carcinoma: incidence and impact. Head Neck.

[52] Yoskovitch A., Hier M.P., Okrainec A., Black M.J., Rochon L. (2001). Skin metastases in squamous cell carcinoma of the head and neck. Otolaryngol Head Neck Surg.

[53] Alwaheeb S., Ghazarian D., Boerner S.L., Asa S.L. (2004). Cutaneous manifestations of thyroid cancer: a report of four cases and review of the literature. J Clin Pathol.

[54] Dahl P.R., Brodland D.G., Goellner J.R., Hay I.D. (1997). Thyroid carcinoma metastatic to the skin: A cutaneous manifestation of a widely disseminated malignancy. J Am Acad Dermatol.

[55] Cohen P.R. (2015). Metastatic papillary thyroid carcinoma to the nose: report and review of cutaneous metastases of papillary thyroid cancer. Dermatol Pract Concept.

[56] Koller E.A., Tourtelot J.B., Pak H.S., Cobb M.W., Moad J.C., Flynn E.A. (1998). Papillary and follicular thyroid carcinoma metastatic to the skin: a case report and review of the literature. Thyroid.

[57] Cheng S.H., Hu S.C. (2020). Skin metastasis from papillary thyroid carcinoma: a rare case with an unusual clinical presentation. Australas J Dermatol.

[58] Hussein M.R. (2010). Skin metastasis: a pathologist’s perspective. J Cutan Pathol.

[59] Tomuleasa C., Zaharie F., Muresan M.S., Pop L., Fekete Z., Dima D. (2017). How to diagnose and treat a cancer of unknown primary site. J Gastrointestin Liver Dis.

[60] Spratt D.E., Spratt E.A.G., Wu S., DeRosa A., Lee N.Y., Lacouture M.E. (2014). Efficacy of skin-directed therapy for cutaneous metastases from advanced cancer: a meta-analysis. J Clin Oncol.

[61] Azemar M., Djahansouzi S., Jäger E., Solbach C., Schmidt M., Maurer A.B. (2003). Regression of cutaneous tumor lesions in patients intratumorally injected with a recombinant single-chain antibody-toxin targeted to ErbB2/HER2. Breast Cancer Res Treat.

[62] Gibbons I.L., Sonagli M., Bertolli E., Macedo M.P., Pinto C.A.L., Duprat Neto J.P. (2018). Diphencyprone as a therapeutic option in cutaneous metastasis of melanoma. A single-institution experience. An Bras Dermatol.

